# Artificial Intelligence Methods in Forest Biotechnology: Current Status and Future Prospects

**DOI:** 10.3390/ijms27104185

**Published:** 2026-05-08

**Authors:** Vadim Lebedev, Andrey Lebedev

**Affiliations:** 1 Branch of Shemyakin-Ovchinnikov Institute of Bioorganic Chemistry, Russian Academy of Sciences, Pushchino 142290, Russia; 2 The Faculty of Fundamental Sciences, Bauman Moscow State Technical University, Moscow 105005, Russia; 2011andreylebedev@mail.ru

**Keywords:** artificial neural networks, forest breeding, genetic engineering, in vitro culture, machine learning, omics technologies

## Abstract

Artificial intelligence (AI) is a field within computer science that is increasingly applied across a wide range of industries. Global climate change and human activity are leading to deforestation, which can have serious ecological and economic consequences. One way to conserve natural forest resources is to create high-yielding and stress-tolerant varieties of tree species with the desired quality characteristics of raw materials using biotechnological breeding methods. In this review, we summarize the achievements and current status of research on the application of AI in forest biotechnology. We examine machine learning algorithms and artificial neural network architectures with respect to their use in various areas of forest biotechnology: in vitro culture, transgenic plants, genome editing, omics technologies, and genomic selection. The review discusses challenges specific to woody plants, such as the deficiency of datasets for model training, as well as the ethical aspects of AI use, including interpretability, bias, and accountability. Finally, we suggest future research directions for consideration. This review may be useful for AI specialists, researchers in plant sciences, forestry practitioners, and policymakers to comprehensively understand the role of AI technologies in investigating and improving forest trees.

## 1. Introduction

Forests are the dominant terrestrial ecosystems. They cover about 31% of the Earth’s land surface and account for about 80% of terrestrial biodiversity [1]. Forests play a significant role in global cycles of carbon, water, and nutrients. For humans, forests serve as a source of construction and fuelwood, cellulose, and various types of raw materials. However, these vital ecosystems face a number of threats. Demand for forestry products is constantly increasing due to both population growth and rising popularity of environmentally friendly materials [2]. Global climate change intensifies the impact of droughts and the spread of pathogens and pests [3]. All of this is leading to a reduction in forest area and threatens their vital ecological and economic roles, therefore requiring urgent action and innovative solutions for their conservation [4].

Sustainable forest management implies a comprehensive approach to the use of forest resources that ensures a balance of ecological, economic, and social goals [5]. The use of artificial intelligence (AI) methods has transformed forest management, primarily by improving the analysis of large and complex datasets obtained through remote sensing [6]. AI is used for a wide range of purposes, including forest inventory and mapping, fire and pest outbreak monitoring, growth and productivity forecasting, and timber resource management [7,8]. This has been made possible by the development of various vegetation indices, which represent numeric indicators of plant quantity and quality and are calculated from two or more spectral bands of remote sensing [9]. Another approach to preventing the loss of natural forests is the creation of forest plantations—intensively managed artificial stands intended to produce specialized high-yield raw materials [10]. Forest species possess significant genetic and phenotypic diversity that can be used in breeding [11]. Unfortunately, traditional breeding methods are very time-consuming due to the long juvenile period of tree species. Over the past 20 years, forest genomics has made significant advances thanks to the emergence of various high-throughput sequencing and bioinformatics analysis technologies. The resulting data can be used to identify genetic markers and genotypes to improve the efficiency of traditional breeding, as well as to identify genes for use in genetic transformation and genome editing [12]. According to Fu et al. [13], there is currently a shift from precision breeding based on multi-omics data and genome editing (Breeding 4.0) toward AI-based breeding (Breeding 5.0). AI methods are capable not only of identifying genetic markers, but also of decoding the architecture and regulatory logic of the genome, as well as its interaction with the environment. Several reviews have been published to date describing applications of AI in crop improvement [14,15,16,17]. To the best of our knowledge, a review study specifically focusing on the use of AI tools in forest biotechnology has not yet been published. This review fills this gap by summarizing the current state of search for genes that determine valuable traits of forest trees (omics technologies), the creation of new tree genotypes (genomic selection, genetic engineering, genome editing) and their efficient propagation (plant tissue culture) using AI methods. The novelty of this review lies in the integration of biotechnology and artificial intelligence for the improvement of perennial woody plants, which are fundamentally different from annual crops in lifespan, structural composition, growth cycle, and ecological impact. The main aim of this review is to provide a comprehensive overview of the application of AI methods in key domains of forest biotechnology. To do this, we (1) review key machine learning algorithms and deep learning architectures, (2) present the existing literature on the current state of knowledge, (3) discuss challenges and (4) suggest future research directions. Ultimately, this study aims to contribute to the development of forest biotechnology to better understand the biology of woody plants and implement sustainable plantation forestry.

## 2. Artificial Intelligence (AI)

### 2.1. Artificial Intelligence Methods

AI is the ability of computer systems to perform tasks that normally require human intelligence, such as learning, problem solving, and decision making [7]. Traditional statistical methods based on assumptions of linearity and a limited number of predictors often prove insufficient when analyzing complex, nonlinear biological systems and high-dimensional datasets [18]. In contrast, AI tools such as machine learning (ML) and deep learning (DL) can handle continuous, binary, discrete, and incomplete data and capture nonlinear, hierarchical, and context-dependent regulatory relationships [19,20].

ML is a subset of AI; its algorithms are trained to solve specific tasks directly from available data [21]. ML methods include supervised learning, which uses labeled datasets for prediction and classification, and unsupervised learning, which uncovers patterns and relationships in unlabeled data for clustering and analysis [22]. Among supervised-learning methods used for classification and regression tasks, widely used algorithms include k-nearest neighbors (KNNs), the Naive Bayes classifier, Support Vector Machines (SVMs), Random Forest (RF), and ensemble methods including gradient boosting algorithms (extreme—XGBoost, categorical—CatBoost), many of which are general-purpose and can be applied to both types of tasks [23]. Unsupervised methods perform two main functions: clustering by grouping similar objects (k-means algorithms, Gaussian mixture models—GMMs), and dimensionality reduction by projecting data into a lower-dimensional space to reduce complexity and reveal latent structure [23].

**Figure 1:**
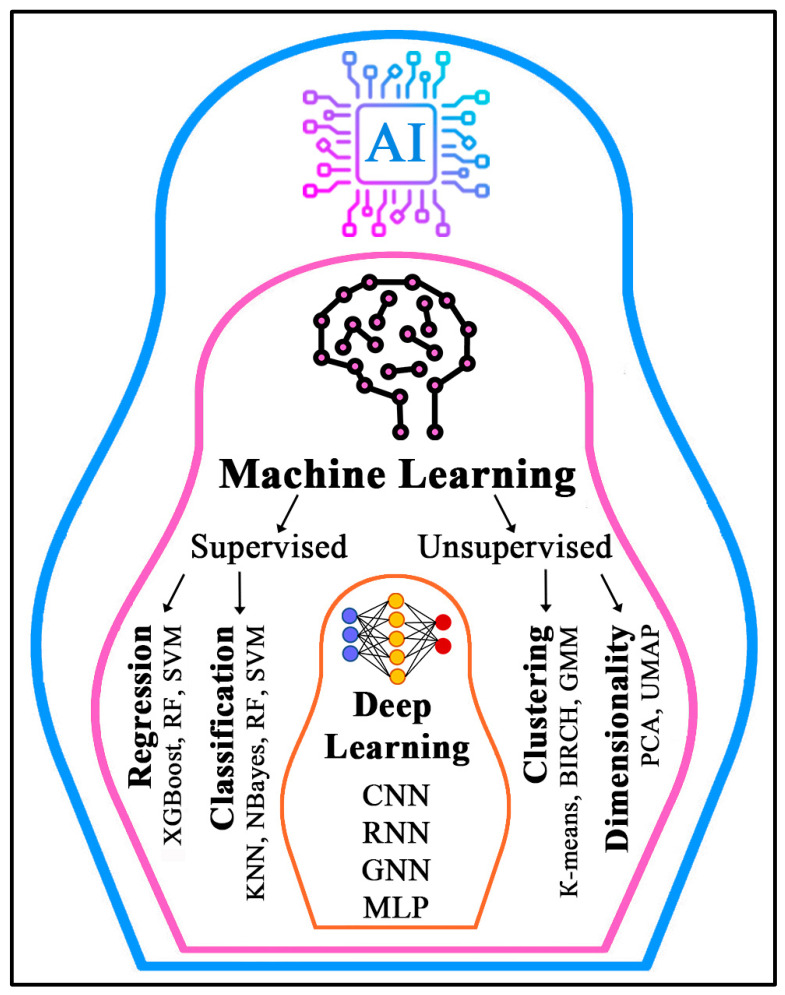
The most common machine learning algorithms and deep learning neural network architectures. AI: artificial intelligence; BIRCH: balanced iterative reducing and clustering using hierarchies; CNN: convolutional neural network; GMM: Gaussian mixture model; GNN: graph neural network; KNN: k-nearest neighbors; MLP: multilayer perceptron; PCA: principal component analysis; RF: random forest; RNN: recurrent neural network; SVM: support vector machine; UMAP: uniform manifold approximation and projection; XGBoost: extreme gradient boosting.

DL is a specialized subset of ML whose methods loosely imitate the operation of biological neurons for learning and decision making; they are well-suited to analyzing large volumes of data but also require such data for effective training [7]. These methods are based on multilayer (“deep”) artificial neural networks (ANNs), in which different nodes (“neurons”) receive input from a lower hierarchical layer [24]. Unlike many traditional ML methods that rely on predefined features, DL methods can automatically extract multilayer representations of data and reveal complex nonlinear dependencies between input and output variables [25]. ANNs differ in their architecture (number and types of layers and connections between them): known types include convolutional (CNN), recurrent (RNN) and graph (GNN) neural networks, multilayer perceptron (MLP), and others [26]. Figure 1 shows the main ML algorithms and DL neural network architectures.

### 2.2. Artificial Intelligence in Plant Biotechnology

ML and DL methods are applied across various areas of plant biotechnology. In vitro culture is a powerful tool for rapid propagation, as well as for improving crops through genetic transformation and genome editing. For maximum efficiency, in vitro culture conditions must be optimized for each species, sometimes for each cultivar, and for different growth and development stages such as proliferation, rooting, somatic embryogenesis, and others [27]. However, due to the large number of components in nutrient media, such optimization is laborious and time-consuming [28]. AI models have become powerful tools for modeling, predicting, and optimizing various in vitro culture systems, including sterilization for culture induction, seed germination, induction of polyploids, composition of media for organogenesis, somatic embryogenesis, androgenesis, and clonal micropropagation [29].

Transgenic plants with various traits have been successfully cultivated worldwide for many years, and the most common method of obtaining them is *Agrobacterium*-mediated transformation. This is a multifactorial process influenced not only by in vitro regeneration parameters but also by *Agrobacterium* cell concentration, duration of inoculation and co-cultivation, and the type and concentration of selective agents and bacteriostatic antibiotics. Optimizing these factors is a complex process because the genetic transformation results (except for model plants) become evident only after a long time—after regenerated plants are tested for T-DNA integration. For example, regeneration of transformants in woody plants can take months [30]. ML algorithms have been used to optimize factors such as *Agrobacterium* strain, optical density of bacterial suspension, co-cultivation duration, selective antibiotic type, and acetosyringone concentration [27,31].

In recent years, genome editing has gained particular popularity as another method of genetic engineering. Various ML and DL models can be used to evaluate sgRNAs, predict on-target and off-target effects in CRISPR/Cas9 editing, optimize protein structures, and improve the efficiency of base and prime editing [19]. It has been shown that AI-enhanced methods improve target specificity, Cas protein efficiency, and in silico validation [32]. Various types of molecular markers in plant biology were initially used to assess genetic diversity, taxonomic classification, heterosis studies, evolution research, cultivar identification, and marker-assisted selection [33,34]. With the development of next-generation sequencing technologies, their most common applications became quantitative trait locus (QTL) mapping, genome-wide association studies (GWAS), and genomic selection (GS) [35]. High-density single nucleotide polymorphism (SNP) markers, which are the most common form of DNA variation, are used for these purposes. Their frequency in plant genomes ranges from one SNP per 16 bp in eucalyptus to one SNP per 7000 bp in tomato [36]. GWAS are used to identify markers closely linked to QTLs of target traits [37]. GWAS typically face challenges such as handling multidimensional data and detecting complex gene–gene and gene–environment interactions; ML and DL methods help address these issues and improve detection of marker–trait associations [38]. ML methods identify nonlinear relationships and interaction effects, while DL techniques facilitate the modeling of complex patterns and relationships. Omics technologies (genomics, transcriptomics, proteomics, metabolomics, etc.) have contributed to the significant progress of plant genetics and breeding. Building on recent advances in chemical analysis and bioinformatics, they have enabled a systems approach to understanding complex interactions between genes, proteins, and metabolites that shape a particular phenotype [39]. These technologies have increased the predictability of the breeding process, reducing the time and cost of developing new varieties with higher yield, stress resistance, and improved nutritional value. At the same time, they generate large volumes of data that require sufficient computational power and suitable statistical models, including AI tools, to process [40]. ML and DL algorithms in genomics are used, in particular, for the analysis of regulatory DNA sequences; in transcriptomics, for the prediction of gene transcription and translation; and in proteomics, for the detection of protein–protein interactions [41,42].

Traditional phenotyping based on visual assessment, manual measurements, and destructive analyses remained a bottleneck in plant breeding for a long time [43]. While high-throughput sequencing was already used for DNA and RNA analysis, phenotype analysis remained laborious, time-consuming, and subjective. The situation changed with the development of automated high-throughput phenotyping (HTP) technologies, allowing for assessment of various plant phenotypic traits from images across different parts of the spectrum [44]. The acquisition of such images and their subsequent analysis led to the emergence of a new omics technology—phenomics, which allow for a better understanding of phenotype formation as a complex interaction of genotype and environment [45]. AI methods, namely computer vision (CV), ML, and DL, are widely used to transform sensor data into quantitative traits and to develop models of genotype–phenotype–environment interactions [46].

Effective implementation of AI methods in plant biotechnology depends on data quality and quantity, as well as the most appropriate algorithm choice. Large and diverse datasets are necessary to train complex models, especially DL architectures [47]. Noise reduction and outlier handling ensure dataset integrity, and splitting data into training, validation, and test sets enables evaluation and fine-tuning [48,49]. The choice of the optimal AI approach depends on the volume and nature of the data and the desired outcome. Sequence-based tasks typically use CNNs or transformers; feature-based analyses favor gradient boosting or RF; structure-based studies use GNNs [19]. Specifically, generalized regression neural networks (GRNNs) and RF algorithms are used to optimize media composition [50], RF and SVM algorithms are used for protein–protein interaction analysis [42], and CNNs are used for image analysis [51]. DL is well-suited to large-scale studies where capturing complex genotype–phenotype relationships is required [24]. Table 1 provides an overview of how AI tools are applied in various aspects of plant biotechnology.

## 3. Artificial Intelligence in Forest Biotechnology

**Figure 2:**
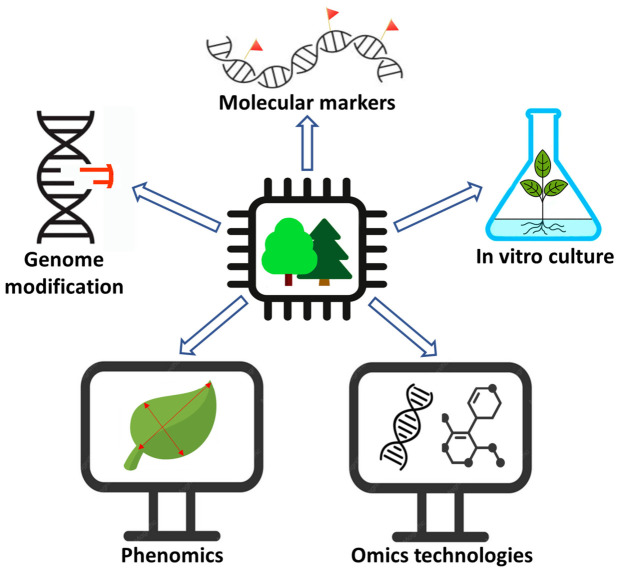
Application of AI in forest biotechnology.

Research involving woody plants often lags behind that on herbaceous species (model organisms or major crops) due to their biological characteristics—large size, long lifespan, and high genome heterozygosity—which increase the labor intensity and complexity of experiments. AI methods are no exception, but ML and DL algorithms have already been used in all areas of forest biotechnology by now (Figure 2). Examples of such studies are shown in Table 2.

### 3.1. Plant Tissue Culture

Plants contain a large number of pharmacologically active compounds used to treat many diseases. The complex structure of some compounds makes chemical synthesis difficult, and extraction from plants is limited by low yields or restricted natural supplies [81]. A promising strategy is the use of biotechnological methods, particularly plant cell cultivation. Paclitaxel, a diterpenoid alkaloid found in *Taxus* species, is one of the most promising anticancer drugs because of its unique mechanism of action [82]. Hazel (*Corylus avellana*) suspension culture is a promising and low-cost strategy for producing paclitaxel, since it is less demanding than *Taxus* culture [25]. Paclitaxel biosynthesis is a complex biological process influenced by many factors and their nonlinear interactions [83]. Experimental optimization of these factors is laborious, expensive, and time-consuming; for this purpose, a number of AI-based studies have been conducted on *C. avellana* cell culture.

To predict paclitaxel biosynthesis, Farhadi et al. [64] compared five regression methods and an adaptive neuro-fuzzy inference system (ANFIS)—a hybrid of fuzzy logic and neural networks that combines the advantages of both methods [84]. Experiments included treating the cell culture with three different elicitors from the endophytic fungus *Coniothyrium palmarum* at various concentrations, alone or combined with methyl-β-cyclodextrin, as well as elicitor addition time and culture harvest time. ANFIS models showed higher accuracy (0.94–0.97) compared to regression models (0.16–0.54). ANFIS model optimization using a genetic algorithm (GA) identified culture conditions for maximal paclitaxel biosynthesis (428 μg/L) [64]. In another study, Salehi et al. [83] compared the effectiveness of regression models and MLP-GA models. Evaluation of the effects of adding fungal cell extract or culture filtrate of *Camarosporomyces flavigenus* during mid and late logarithmic growth phases showed higher accuracy for MLP-GA models (0.89–0.95) compared to regression models (0.56–0.85). The MLP-GA model, developed in this study, could accurately predict dry weight, biosynthesis, and secretion of paclitaxel in *C. avellana* cells [83]. Using the same experimental conditions, Salehi et al. [25] predicted paclitaxel biosynthesis with a GRNN and fruit fly optimization algorithm (FOA). Optimization analysis on the GRNN-FOA models showed that paclitaxel yields could reach 373 μg/L.

Development and optimization of culture media composition is a major application of AI in plant culture in vitro. Mineral composition is the basis of any medium, and nitrogen is the most important element. Optimal total nitrogen concentration and nitrate–ammonium ratio in the medium depend on tissue type, genotype, and cultivation conditions [85]. To optimize nitrogen content for organogenesis of *Pinus taeda* L., Barone [65] created two models: a parametric method (multiple regression analysis) to determine linear relationships between variables, and a nonparametric method (ANN) to reveal nonlinear effects. Model testing showed that both total nitrogen and nitrate–ammonium ratio affected morphogenesis, but the ANN demonstrated higher accuracy in predicting morphogenetic responses [65].

Somatic embryogenesis is the preferred method for the propagation of conifers in vitro, but somatic embryo size, large variation in cotyledon number and their overlap complicate traditional image analysis methods based on pixel intensity differences [86]. DL methods using CNNs can accurately distinguish different objects within an image and are resistant to noise defects [87]. Davidson et al. [66] were the first to use CNNs to analyze mature somatic embryos of *Pinus radiata* D. Don. The Mask R-CNN model effectively segmented embryos from proliferating embryogenic tissue and counted cotyledon numbers. Besides high accuracy in selecting suitable embryos, this method reveals complex patterns in large datasets, which can optimize culture conditions and improve somatic embryogenesis efficiency [66].

Synthetic seeds are somatic embryos or other vegetative reproductive organs obtained in vitro and coated with special substances containing nutrients and protective compounds. Kocak et al. [67] encapsulated nodal segments of hawthorn *Crataegus monogyna* and predicted regeneration of synthetic seeds under the influence of indole acetic acid (IAA), storage temperature (−20, +4, +24 °C) and duration (1, 2, 3 months). Five ML algorithms were used for analysis: Decision Tree (DT), Gaussian process (GP), MLP, RF and XGBoost. Four of the five models showed high predictive accuracy (97.3%). DT and XGBoost models identified temperature as the most significant factor, while MLP and RF models indicated IAA [67].

### 3.2. Genome Modification

Plants adapt to water deficit through various morphological, physiological, and biochemical responses. One way to increase drought tolerance is the accumulation of osmolytes, including ammonium compounds, sugars, sugar alcohols, and amino acids [88]. To assess drought tolerance of two poplar clones (*Populus tremula* × *P. tremuloides*) carrying the *AtGolS2* galactinol synthase gene in the field, Shikakura et al. [68] used ML methods. Eighteen months after planting, plants were subjected to drought for 100 days by covering the soil with film, and chlorophyll fluorescence quantum yield was measured twice every week. Photosynthesis and soil moisture data were clustered using GMM and k-means models. Both models divided the data into three groups, but GMM was preferred, as it identified soil moisture as the primary factor, whereas k-means highlighted quantum yield. Quantum yield did not differ significantly between genotypes under moderate drought (soil moisture of 30–47%), but under severe drought (<30%) it was significantly higher in the transgenic clones [68]. Jalil et al. [89] evaluated *Populus alba* plants for tolerance to salinity. Three transgenic clones expressing the *aqua1* aquaporin gene and a control were grown for 28 days under salinity and non-salinity conditions (100 and 0 mM NaCl). Red–green–blue (RGB) leaf images were analyzed using a CNN-based DL method, and ML algorithms (RF, SVM, and logistic regression (LR)) were used to detect plants treated with 100 mM NaCl. Classification using SVM and RF reached maximum accuracies of 75% and 71%, respectively [89].

Synthetic promoters that provide precise temporal and spatial control of transgene expression in transgenic plants are among the most advanced tools of synthetic biology [90]. To identify potential transcription factor binding sites in promoters of all genes of *Populus trichocarpa* and *P. tomentosa*, Lu et al. [91] analyzed transcriptomic data with RF models that accounted for tissue-specific expression. Based on discovered root-specific cis-regulatory modules, two synthetic promoters, PRTS1 and PRTS2, containing 21 and 46 transcription factor binding sites respectively, were designed. For validation, binary vectors carrying the synthetic promoters fused to the *uidA* reporter gene were used for *Agrobacterium* infiltration of poplar seedlings. The PRTS1 promoter demonstrated strong root-specific GUS expression and can be used for targeted gene expression in forest biotechnology [91].

The efficiency of extracting cellulose fibers from wood is determined by lignin properties, which binds cellulose and hemicellulose in plant secondary cell walls [92]. Modifying lignin content and/or composition is a major aim of forest biotechnology, and many transgenic trees with altered expression of individual lignin biosynthesis genes have been produced by now [93]. To target multiple lignin biosynthesis genes simultaneously, Sulis et al. [69] used multiplex CRISPR editing in *Populus trichocarpa*. Using a predictive model based on ML algorithms, they analyzed nearly 70,000 multi-gene editing variants for 21 lignin biosynthesis genes and ultimately selected seven strategies aimed at simultaneous modification of up to six genes. The model predicted lignin content reduction of up to 35% and S-G and carbohydrate–lignin ratios increases of up to 248% and 215%, respectively, without affecting tree growth [69]. Delivery of the CRISPR construct via *Agrobacterium tumefaciens* resulted in 174 edited lines. After six months in the greenhouse, some genotypes showed that lignin content reduced to 50% and the carbohydrate–lignin ratio increased to 228%. Multiplex editing guided by ML models substantially reduced lignin content, which should increase cellulose yield, improve the environmental profile of production, and reduce greenhouse gas emissions [69].

### 3.3. Molecular Markers

#### 3.3.1. Genetic Identification

Genetic identification methods can be used to determine the population membership of individual trees or groups of individuals [94]. Molecular markers such as SNPs and simple sequence repeats (SSRs) have proven highly effective for determining population structure and are widely used to study genetic diversity in plant species [95]. For the classification of *Eucalyptus cladocalyx* subpopulations, Maldonado et al. [96] used several methods: partial least squares discriminant analysis (PLS-DA), CNN and MLP. For 310 trees from 49 half-sib families, leaf reflectance spectra and genotyping data (~60,000 SNPs) were obtained. The MLP model showed the highest accuracy for classifying individuals by subpopulation (87%). This method demonstrated robustness with imbalanced datasets and can be applied to other forest tree species [96].

Genetic adaptation of trees to local environments is becoming important with global climate change, when reproductive material must originate from regions matching current and future conditions at restoration or plantation sites [97]. To determine the origin of *Pinus pinaster*, Olsson et al. [98] analyzed 10,185 SNPs from 1579 samples collected from 86 populations, 45 regions and 10 gene pools covering the species’ range. They used RUBIAS v0.3.3 software for genetic identification and the assignPOP ML method for origin assignment. Both programs showed very similar accuracy and indicated that origin assignment for *P. pinaster* is reliable at the gene-pool and regional levels but less so at the population level [98]. To predict the geographic origin of deciduous tree species, Degen et al. [70] compared best linear unbiased prediction (BLUP), nearest neighbors, Gaussian process regression (GPR) and feedforward neural network (FFNN) methods. They used 30,000 SNPs from 865 European beech (*Fagus sylvatica*) trees and 381 SNPs from 1883 oak (*Quercus robur*) trees sampled across Europe. FFNN was the best method for both datasets and was able to learn patterns associated with different geographic origins automatically [70].

Illegal logging is a major problem causing forest loss and biodiversity decline. One way to control it is to trace timber origin using identification methods: visual (macro- and microscopy), chemical (chromatography and mass spectrometry) and genetic (DNA analysis) [99]. Among these, DNA barcoding of timber (labeling based on DNA sequence) provides high identification accuracy and protection against falsification [100]. To analyze the DNA barcode database, Dev et al. [101] used the ML WEKA package. In addition to *rbcL*, *matK* and *psbA-trnH* gene regions for 41 commercially valuable timber species from southern India, anatomical wood traits were included in the analysis. Samples were classified using four algorithms—SVM, RIPPER, DT and Naive Bayes—with SVM showing the highest accuracy [101]. Studying population genetic structure with molecular markers is important for understanding ecological and evolutionary dynamics of forest species, as well as for applied purposes including law enforcement and GWAS [96].

#### 3.3.2. Genome-Wide Association Studies

Genome-wide association studies (GWAS) aim to analyze hundreds of thousands of SNP markers and detect those associated with specific traits [102]. Because of linkage disequilibrium there can be many candidate genes, and various ML methods are used to prioritize them and provide robust genotype–phenotype association detection [103].

Adventitious root and shoot formation is critical for the success of clonal micropropagation (shoot rooting) and the regeneration of transgenic plants. Regeneration ability is highly genotype-dependent and is controlled by endogenous and exogenous factors, while its molecular basis is still poorly understood. Roots can be phenotyped not only by linear size but also by branching pattern and/or origin [104]; calli and shoots show high variability and complex shapes, colors and sizes [105], so several CV methods have been developed for their image analysis. To identify genetic regulators of adventitious rooting, Nagle et al. [104] performed GWAS using 1148 *Populus trichocarpa* genotypes. For the measurements, a DeepLab network with ResNet50 as a backbone was developed. Two segmentation models were used: one separating plants from background, and another segmenting plants into leaves, stems and roots. Using ~34 million SNPs, they found 277 unique associations linked to genes involved in hormone signaling, cell division, reactive oxygen species signaling and other processes with known effect on root development. Results indicate that root formation is a polygenic trait; however, the effects of most genes are insignificant [104].

The same poplar population was later used to identify genes responsible for shoot regeneration [105]. Callus and shoot regeneration were evaluated on cuttings *in planta* from sequential RGB images using a CV method based on a deep CNN with PSPNet architecture. Over 200 candidate genes were identified, including regulators of cell adhesion, stress responses and hormonal signaling pathways, suggesting that regeneration is affected not only by auxins and cytokinins but also by jasmonates and salicylic acid [105]. Fluorescence hyperspectral imaging was later added to the RGB-based method to use fluorescent proteins as markers of tissue transgenic status [106]. *Populus trichocarpa* cuttings were transformed using *Agrobacterium* with a plasmid containing the *eGFP* (green fluorescent protein) gene. The new algorithm segmented RGB images into callus, shoot and nonregenerating stem, and analysis of fluorescence hyperspectral data with CubeGLM detected GFP signal. More than 400 candidate genes were identified, many of which were transcriptional regulator genes and protein–protein interaction genes, indicating a complex gene regulatory system controlling the regeneration of transgenic tissues [106].

To improve tree productivity, a search was conducted for genes associated with leaf venation, which supports leaf mechanical structure and regulates water and assimilate distribution [107]. For lamina and visible vein segmentation, Lagergren et al. [108] used few-shot learning based on CNNs, requiring only a small number of samples. From 2906 field images of *Populus trichocarpa* leaves, 68 traits related to leaf, vein and petiole morphology were extracted. Sequencing of 1492 trees produced 847,066 SNPs. GWAS was used to analyze vein density, as this parameter balances light capture and carbon fixation with sugar and nutrient transport. A total of 30 unique genes usable in molecular breeding for climate resilience and biomass production were identified [108]. In order to improve tree productivity, another study investigated photosynthesis, which directly affects biomass accumulation. Jiang et al. [71] performed GWAS on 300 unrelated *Populus tomentosa* individuals from a natural population using 2,024,059 SNPs and 17 traits grouped into photosynthetic parameters, pigment content and enzyme activity. Five ML algorithms (XGBoost, RF, support vector regression (SVR), least absolute shrinkage and selection operator (LASSO) and KNN) were used, with SVR giving the best predictions. The study identified the *PtoIRKI* gene, whose protein enhances RuBisCO activity, increasing photosynthetic efficiency and starch accumulation in chloroplasts, and an elite haplotype with elevated expression of this gene [71].

The pathogenic fungus *Hymenoscyphus fraxineus* invaded Europe from Asia in the 1990s, causing common ash (*Fraxinus excelsior*) mortality [109]. To identify resistant genotypes, Doonan et al. [110] conducted GWAS on 486 trees from six European countries. They searched for associations between SNP markers and resistance phenotypic traits using RF, revealing eight SNPs associated with crown damage and located in genes related to plant defense responses and phenology. These markers can help identify resistant ash genotypes for breeding or reforestation [110].

#### 3.3.3. Genomic Selection

Breeding cycles of forest species vary from 15 years in eucalyptus [111] to 30 years in conifers [112]—and such long periods limit their genetic improvement. Genomic selection (GS), unlike marker-assisted selection, uses large numbers of DNA markers to predict genotype breeding value and can greatly accelerate this process [113]. Traditional prediction methods are based on parametric models (BLUP, Bayes), which may not capture complex genotype–phenotype relationships [114]. ML and DL use nonlinear models, thus improving prediction accuracy. One of the main fields in GS of forest species is productivity improvement. To conduct GS for the stem circumference of rubber tree (*Hevea brasiliensis*), Aono et al. [115] evaluated the GS effectiveness of two classical statistical models (Bayesian ridge regression (BRR) and a single-environment, main genotypic effect model with a Gaussian kernel (SM-GK)) and four ML algorithms (AdaBoost, MLP, RF and SVM). A new approach was proposed: the use of neural networks for two-stage prediction, first predicting subpopulation and then phenotype. For two tree groups genotyped with SNP (107,466) or SSR (332) markers, ML algorithms outperformed traditional models [115]. For GS for growth traits of commercially important tropical species, *Shorea macrophylla*, Akutsu et al. [116] tested 12 models: six classical (genomic BLUP (GBLUP), BayesA, BayesB, BayesC, LASSO-B, BRR) and six ML (reproducing kernel Hilbert space regression (RKHS), RF, XGBoost, light gradient boosting (LGB), CNN1D and CNN2D). They identified 18,037 SNPs and measured the height and diameter of trees. With the full SNP set, RF gave the best results; with reduced sets (48, 96, 192 SNPs) selected by GWAS, GBLUP, RKHS, LGB and CNN1D performed best [116].

Traits of forest trees can change over years, but this temporal aspect is rarely addressed in GS. Zhou et al. [117] performed GS for growth and wood properties in *Populus deltoides*, measuring height and diameter annually for four years in 765 hybrid trees. Wood density, microfibril angle, fiber length and width were also measured on cores. About 1.14 million SNPs were identified. Fourteen models were used: two BLUPs (RR-BLUP, GBLUP), four Bayesian (BayesA, BayesB, BayesC, BRR), seven ML (gradient boosting decision tree (GBDT), RF, kernel ridge regression, ridge regression, elastic net regression, MKRKHS, Light gradient boosting machine) and a DL model (deep neural network Gaussian process) [117]. GWAS identified 135 SNPs associated with growth and wood traits. Bayesian and BLUP methods had similar predictive ability, while ML models better predicted complex low-heritability traits. Traditional methods (RR-BLUP, BayesB) were optimal for diameter and wood density, while ML methods (RF, GBDT) were superior for height, microfibril angle, fiber length and width [117]. The same *P. deltoides* population and model set were used in Zhou et al. [118] to predict sex of trees. Early sex identification in poplar is important because breeders prefer male cultivars due to several environmental issues caused by female trees. The GBDT ML model provided the best accuracy across all marker densities but required the longest computational time.

*Eucalyptus globulus* is a key source of essential oil used pharmaceutically because of high monoterpene 1,8-cineole content (up to 90%) [119]. To predict leaf essential oil content, trunk quality and growth traits, Mieres-Castro et al. [72] evaluated two DL models (CNN and MLP) using uni- and multi-trait approaches. A breeding population of 1968 *E. globulus* (65 families) was used. Phenotypic traits (leaf near-infrared (NIR) absorbance, trunk height, diameter, branching, straightness and volume) were measured at the age of 9 years. Genotyping used 14,442 SNPs. DL-based prediction models showed higher accuracy with multi-trait approaches for most traits [72]. Another eucalyptus study predicted dry condition adaptation traits. Mora-Poblete et al. [114] compared classical models (BayesA, BayesB, BayesC, BL, BRR) and DL models (CNN, MLP) on 310 *Eucalyptus cladocalyx* trees (49 half-sib families) from five Australian populations using SNP and phenotype data (height, diameter, trunk volume, etc.) measured at an interval of four years. DL models outperformed Bayesian models for most traits [114]. Thus, AI models can substantially increase predictive accuracy in programs of forest tree genomic selection.

### 3.4. Omics Technologies

Poplar (*Populus*) is a model plant for studying tree genomics, and its hybrid forms are widely used in forestry plantations because of their high productivity. In particular, the hybrid 84K (*P. alba* × *P. tremula* var. *glandulosa*) from South Korea is valued for its fast growth and high adaptability [120]. The genetics of heterosis, where hybrids outperform their parents, is still not fully understood [121]. Allele-specific expression (ASE) is one possible mechanism of heterosis, and to study it, Shi et al. [73] used high-accuracy and ultra-long DNA sequencing to assemble two haplotype subgenomes of the 84K hybrid, and RNA sequencing to identify and annotate genes. ML XGBoost models based on 46 genetic and epigenetic features were used to predict ASE groups and identify key factors influencing it. These models showed that CHG methylation in the gene body, sequence divergence and the presence of transposons both upstream and downstream of alleles are important factors for ASE [73].

Understanding wood formation processes has both fundamental and applied significance. To study secondary vascular tissue, which is important for understanding the radial growth of trees, Du et al. [122] combined high-resolution anatomical analysis of stems from two-month-old 84K poplar hybrid plants with spatial transcriptomics. Expression of 2000 genes in six representative stem tissue sections was analyzed using the uniform manifold approximation and projection (UMAP) ML algorithm. In total, 17 distinct clusters corresponding to different developmental stages were identified and classified into metaclusters of primary and secondary growth [122]. Long noncoding RNAs (lncRNAs) are associated with secondary cell wall biosynthesis, which determines the physical and mechanical properties of wood, and to study them Wang et al. [123] used 231 bamboo (*Phyllostachys edulis*) RNA-seq datasets. The RF algorithm was applied to improve genome-wide identification of lncRNA candidates and functional annotation. In total, 37,009 lncRNAs were identified and functions for more than 65% of them were successfully annotated. In the PAL/4CL/C4H gene families, one gene predominantly encoding lignin and four genes predominantly encoding flavonoids were identified, which is important for molecular breeding [123]. To test the hypothesis that cytosine methylation can be used to assess physicochemical wood traits, Champigny et al. [74] estimated 25 *Populus balsamifera* genotypes from nine provenances grown at two field sites. Methylation intensity was measured in leaf and xylem samples, and associations between CpG methylation and poplar traits were modeled using FFNN. Significant proportions of phenotypic variance in poplar biomass, wood density, lignin, and mannose contents were explained by epigenetic variation. The authors believe that epigenome-based models have significant potential in validating the identity, origin, and quality of forestry products.

Global climate change increases the frequency and intensity of drought periods, which reduces forest productivity and increases tree mortality. Plant response to drought is a complex mechanism involving many protective genes, proteins, and signaling pathways [124]. To identify genes that are responsive to water stress, Tahmasebi et al. [75] analyzed data from 13 studies with various poplar species subjected to drought. Meta-analysis and ML algorithms, including SVM and others, were used to analyze large volumes of transcriptomic data. The study showed that transcriptional changes in poplar under drought can be extremely diverse. As a result, key signaling pathways and genes were identified, in particular ARF2-like and PYL4-like types, which can be used as markers in breeding woody species for drought tolerance [75].

A gene regulatory network visually represents complex regulatory interactions between regulators and their target genes that together provide growth, development, and adaptation of plants to various stresses [125]. Traditional experimental methods, including yeast one-hybrid (Y1H), chromatin immunoprecipitation sequencing (ChIP-seq), and DNA affinity purification sequencing (DAP-seq), are labor-intensive and low-throughput; Mummadi et al. [20] proposed predicting gene regulatory networks using AI. The study compared four supervised learning models—LR, SVM, DT, KNN—and five ensemble learning models—RF, Extremely Randomized Trees, AdaBoost, Gradient Boosting, and Bagging Classifier. *Populus trichocarpa* transcriptomic data were used for evaluation. LR, SVM, and Bagging showed lower average accuracy (77–79%) compared to other models (88–97%), and ensemble methods were more effective. Hybrid models combining DL (CNN) and ML identified a larger number of transcription factors involved in lignin biosynthesis and showed strong prioritization capabilities [20].

Orphan genes, which have no homologs outside their species, play an important role in plant stress responses [126]. Drought and high temperature affect the productivity of bamboo (*Phyllostachys edulis*), and Zhang et al. [76] used 10 different ML and DL models (RF, SVM, RNN, etc.) to predict orphan genes. Homology searches of 31,987 bamboo proteins among protein sequences from 136 other plant species showed that 1936 proteins were unique. Ultimately, 1544 bamboo orphan genes were identified, and a new hybrid DL model based on convolutional and transformer neural networks (CNN + Transformer) demonstrated superiority across all four composite evaluation metrics [76].

AI models trained on plant metabolome data are relatively rare, likely due to the high cost of obtaining large datasets needed to train models [23]. Beneficial endophytes help plants survive under stressful conditions by altering host metabolism, and Aufrecht et al. [77] evaluated the effect of inoculation with a consortium of nine strains on the metabolic network of *Populus trichocarpa* under water deficit. In rooted cuttings, the effects of inoculation and drought on intracellular (roots) and extracellular (root exudates) metabolomes were assessed using three ML approaches: LR, KNN, and DT. KNN models performed best, achieving 100% accuracy in prediction. It was found that endophytes spatially alter the root cell metabolome depending on their type and location. Using ML models, metabolites in roots and exudates were identified that may serve as indicators of drought and inoculation status by endophytes [77].

### 3.5. Phenomics

Phenomics is also an omics technology but, unlike others, it analyzes images rather than instrumental data. Image acquisition (high-throughput phenotyping, HTP) is performed at multiple scales: microscopic, ground (mobile or stationary platforms), and aerial (unmanned aerial vehicles (UAVs), airplanes, satellites). Standard software is used to analyze changes in individual traits (e.g., in transgenic plants) [127,128], but AI methods are employed to process large datasets or detect stress responses.

#### 3.5.1. Microscopic High-Throughput Phenotyping

Stomata are microscopic pores on the plant epidermis formed by a pair of guard cells; they play a key role in regulating gas exchange and transpiration [129]. These processes are influenced by stomatal density, stomatal size, and pore size [130]. To analyze stomata of *Populus simonii* × *P. nigra* and *P. lasiocarpa*, Li et al. [131] used 1000 microscopic images. Stomata were successfully detected with Faster R-CNN, a DL architecture for object detection in natural images. Song et al. [132] used another CNN method, Mask R-CNN, for stomatal segmentation and parameter measurement. It automatically measured stomatal pore anatomical parameters on images of *Populus nigra* leaves. Later, Mask R-CNN was applied to detect stomata across many woody species including poplar, birch, ginkgo, 27 eucalypt species, and 10 gymnosperm species [78]. More than 2800 images were used, and the method provided robust image quality regardless of plant species, sampling and imaging methods, and magnification level. Mask R-CNN allows for direct measurement of stomatal orientation, axis lengths, and total area without additional image processing steps [78]. Gibbs et al. [133] used CNNs to estimate stomatal conductance (gas exchange rate), which depends on stomatal density and size. The DL model based on Attention U-Net and Inception architectures was tested on two datasets with different stomatal shapes: the dicot poplar (*Populus balsamifera*) and the monocot wheat (*Triticum aestivum*). The method achieved 100% accuracy in distinguishing stomata between species and automatically computed counts, density, morphological traits, and stomatal conductance. This approach can be used in breeding programs for drought-resistance, which is becoming the main threat to forest stands in the context of global climate change [133].

To determine morphological and chromatic parameters of extremely small (2–3 × 0.6–1 mm) seeds of poplars for breeding programs and genetic studies, Wang et al. [134] developed a vibration-assisted machine-vision system incorporating lighting from alternating directions. Using this system, they obtained over 1,187,000 images of singular seeds from seven poplar cultivars common in China. Image classification was performed with an SVM algorithm, which achieved 0.819 accuracy using only morphological measurements and 0.856 accuracy with morphological and color features combined.

#### 3.5.2. Ground High-Throughput Phenotyping

Timely and accurate assessment of drought stress based on the whole tree phenotype is necessary for germplasm screening when searching for tolerant forms and studying drought tolerance, as well as for forest monitoring. Fan et al. [135] used ML to analyze responses of two poplar cultivars with differing drought tolerance to varying water stress levels in a greenhouse. Seven vegetation indices calculated from multispectral camera images on a phenotyping platform were used, together with morphological measurements and biochemical data. RF was used first to classify cultivars and then to classify drought tolerance. This method detected and evaluated drought-related traits at early growth stages with high accuracy (>95%) [135]. Two other studies used four poplar cultivars with varying drought tolerance. Zhou et al. [136] divided plants into five irrigation groups and used RGB photos to estimate leaf posture and drought degree. A combination of CV and DL was used: Mask R-CNN and YOLOv8 for leaf segmentation, and CNNs (single-task and multitask) for simultaneous stress level and cultivar classification. The multitask MobileNet achieved the highest performance (99% for cultivar identification and 76% for stress level classification), outperforming commonly used single-task DL models [136]. In Zhou et al. [137], plants were grown in a greenhouse under three treatments (control, moderate and severe drought) and imaged in visible and thermal infrared ranges. RGB images provided vegetation indices, and TIR images provided temperature features. Among ML algorithms including RF, XGBoost, CatBoost, DT, and GBDT, the CatBoost model built on merged feature layers performed best [137].

Ground HTP studies have also been conducted on birch. To identify and classify drought stress in *Betula luminifera* seedlings for selection of tolerant genotypes, Gao et al. [79] proposed a DL model. Six-month-old *B. luminifera* seedlings (two families from different regions) in a greenhouse were drought-treated and over 20,000 RGB photos were acquired. Four classic CNNs (AlexNet, VGG16, GoogLeNet, ResNet) were tested. ResNet50 performed best and was used to develop a new architecture, spatial attention module (SAM)-CNN, which reached 99.6% accuracy. SAM-CNN uses a spatial-attention mechanism and effectively analyzes images with large background content [79]. Leaf detection and counting are important in plant phenotyping; and for this purpose Guo et al. [138] presented an optimized CV YOLOv8 model—MAF-YOLOv8. The model integrates MobileViT architecture and an Adaptive Feature Pyramid Network and accurately counts leaves under varying conditions (lighting changes, noise). It was tested in a greenhouse on four-month-old seedlings from 10 *B. luminifera* families of different origin. Analysis of RGB images showed that MAF-YOLOv8 achieved a mean accuracy of 91.7% in leaf counting [138].

Drought-resistance evaluation has also been applied to conifers, which are important in forestry. About 800 two-year-old *Pinus sylvestris* seedlings from three populations with distinct ecological and geographic origins were split into two groups; one underwent an 84-day drought in a greenhouse [139]. Chlorophyll fluorescence, thermal imaging, top and multiple angle side views were acquired using a HTP platform providing RGB and VNIR/SWIR hyperspectral imaging. K-means was used to segment hyperspectral data; linear discriminant analysis (LDA) and RF were used to classify seedlings based on their origins. LDA achieved higher prediction accuracy of the population origin (78–81%) compared to RF (62–69%), while recognition of the irrigation treatment was similar for both models (54–88% and 54–83%, respectively).

In HTP, visible-light (RGB) and hyperspectral imaging are most commonly used; other sensors are less frequent. To study biochemical changes in *Quercus robur* leaves through the process of autumn senescence, Clark et al. [140] combined Raman spectroscopy, UV spectrophotometry, and RGB imaging. An artificial neural network classified spectral data, while K-means and RF were used for sample classification. The study identified chlorophyll catabolites and other biochemical markers characteristic of senescence stages, with potential applications being in the pharmaceutical and cosmetic industries.

#### 3.5.3. Aerial High-Throughput Phenotyping

AI methods have also been applied to remote-sensing images analysis. To assess water stress at the single-tree level for accelerated selection for drought tolerance and for individualized irrigation strategies, Tauro et al. [141] used latent heat flux (λET, W/m^2^) and a multi-platform approach. A breeding population of 4600 *Populus nigra* trees was grown for two years under two irrigation regimes (drought and control). Images were obtained from the Copernicus Sentinel-2A satellite (multispectral) and UAVs (thermal infrared (TIR) and RGB), while moisture was measured with field sensors. As satellite imagery had lower resolution than UAV imagery, a Data Mining Sharper ML algorithm was used to enhance satellite images and allow for individual tree identification. λET was shown to vary significantly between trees under irrigation and drought and decreased with reduced soil moisture in the root zone [141].

For some species, variation in ploidy level among individuals is a key component of genotypic variability, affecting size, secondary metabolite synthesis, and abiotic stress tolerance [142]. Multiple gene copies can also increase heterosis potential in hybrids [143]. Laboratory ploidy assessment is laborious, limiting study of polyploidy in the context of biogeographic conditions or genotype selection. *Populus tremuloides* is an example of species where individuals of different ploidy coexist [144]. To determine ploidy levels in stands, Blonder et al. [145] combined SSR-marker analysis in samples with UAV remote sensing. Normalized difference vegetation index (NDVI) was computed from multispectral images, and the RF model successfully predicted ploidy level.

Forest diseases are major biotic stresses; timely and accurate detection is critical for their control. Ink disease, caused by *Phytophthora cambivora*, threatens chestnut (*Castanea sativa*) stands in Europe, and Arcidiaco et al. [80] used UAV HTP to detect it. Eight vegetation indices relevant to vegetation status and stress detection were calculated from multispectral imagery. Crown classification (healthy/diseased) was performed with SVM, Gaussian Naive Bayes (GNB), and LR. Best results (95.2%) were obtained using green NDVI and red-edge NDVI indices combined with SVM and GNB classifiers. Visual validation by phytopathologists confirmed the potential of UAV HTP integrated with ML for forest-disease detection [80].

Through photosynthesis, plants convert CO2 into organic matter, producing gross primary production (GPP), the main carbon flux in terrestrial ecosystems. Accurate GPP estimation is important for carbon balance, climate change, and ecosystem dynamics [146]. To build models improving GPP prediction accuracy, Wang et al. [147] used solar-induced chlorophyll fluorescence in *Populus nigra* stands and ML methods. An automatic data-collection system was installed on a tower 18 m above the canopy; five vegetation indices were calculated from collected data. Traditional ML models (RF and back propagation neural network (BPNN)) and their integrations with an MLP (BP/MLP and MLP-RF) were used. Both BP/MLP and MLP-RF showed higher accuracy than BPNN and RF, with BP/MLP consistently outperforming MLP-RF [147]. Overall, AI application in forest-species phenomics at multiple scales enables screening genotypes for stress tolerance for use in breeding, as well as the study of large-scale processes in forest ecosystems.

### 3.6. Usage of Artificial Intelligence Tools

**Figure 3:**
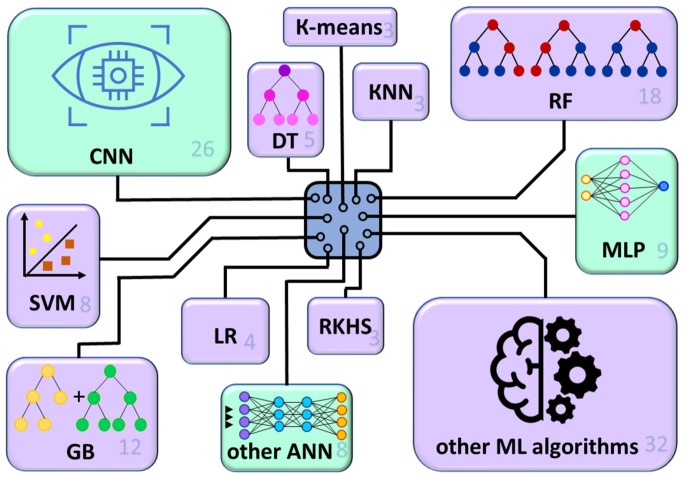
Distribution of the ML algorithms and DL neural networks for forest biotechnology studies in the reviewed publications. Rectangle size reflects the frequency of algorithm usage (indicated in the lower right corner) across publications (50), and colors denote ML (magenta) or DL (green) tools. ANN: artificial neural network; CNN: convolutional neural network; DT: decision tree; GB: gradient boosting; KNNs: k-nearest neighbors; LR: logistic regression; ML: machine learning: MLP: multilayer perceptron; RF: random forest; RKHS: reproducing kernel Hilbert space regression: SVM: support vector machine.

An analysis of the prevalence of AI tools in the reviewed studies reveals a predominance of ML algorithms over ANN (two-thirds versus one-third) (Figure 3). Among the algorithms, the largest contribution is made by RF, as well as the family of gradient boosting models. It is worth noting the wide variety of ML algorithms, which appear only once or twice. Among neural networks, the dominant one is the CNN, which is widely used in CV systems.

## 4. Challenges of Artificial Intelligence

Although AI has great potential in forest biotechnology, there are several technical and ethical challenges that limit its use. One of the main issues is the quality and availability of data on forest trees, as AI models require large, high-quality genomic and phenotypic datasets [148]. Tree species have large and complex genomes; for a number of non-model species, reference genomes are lacking, and phenotypic data are significantly scarcer than for crops. Obtaining new data is also challenging, as tree genomes have a high degree of heterozygosity, and their long lifespan has facilitated the development of epigenetic mechanisms for adaptation to changing environmental conditions. Ultimately, this leads to complex patterns of genotype × environment interactions, making it difficult to understand the relationships between genes of woody plants and its phenotypic traits. Full information on the life cycle of crops can be obtained in a few months of greenhouse cultivation. In contrast, obtaining data for training models or evaluating new genotypes of forest trees requires long-term and expensive field trials. This increases the cost of applying AI to woody plants, while the limited dataset increases the likelihood of unreliable predictions. Finally, the lack of standardization in data formats is a challenge for plant biotechnology in general [149]. This leads to errors when training AI models on data of the same type (e.g., tissue culture) from different sources and integrating data from different fields (e.g., genomics, proteomics and metabolomics).

The integration of AI into forest biotechnology also raises a number of ethical challenges that remain underexplored. A key concern is the interpretability of models, as many AI systems operate as “black boxes,” receiving data and making decisions [149]. Intermediate calculations remain hidden, preventing the validity of large-scale biological data analysis and reducing researchers’ confidence in the accuracy and reliability of the results. Interpretability is also important for identifying and addressing errors in AI models [150]. Algorithmic bias is another concern. Bias in AI models is defined as models created based on biased assumptions or trained on poor-quality datasets, leading to systematically discriminatory results [151]. For example, models used in forensic analysis to combat illegal logging can lead to erroneous conclusions regarding the origin of timber [152]. The two aforementioned issues raise the accountability problem for erroneous decisions made by AI systems. For example, the creation of transgenic or genome-edited trees could lead to long-term environmental impacts or unintended genetic changes. It is currently unclear who will be responsible for such errors: the developer of the AI models, their user, or regulatory authorities [153]. Furthermore, there is the issue of data ownership, such as tree genome information, which is used without proper consent for commercial purposes. In general, the issues of ownership and patentability of intellectual property created by AI are controversial, since existing laws imply that the inventor is a person, not a program. Finally, there is the challenge of the digital divide, where the high cost of using and training AI systems limits their access to researchers in developing countries or institutions with limited budgets [148].

To address these ethical challenges, it is proposed to apply the principle of transparency, which includes the openness, explainability, and understandability of AI systems for all stakeholders: researchers, independent experts, regulators, policymakers, and others. This principle is mentioned in the world’s first law regulating AI [154]. This EU AI Act establishes that the use of AI can contribute to “the conservation and restoration of biodiversity and ecosystems, and climate change mitigation and adaptation,” which aligns with the goals of forest biotechnology. To support innovation, it does not apply to AI systems and models specifically developed solely for scientific research. On the other hand, this law recognizes biological risks as systemic threats, particularly AI models that use biological sequences as inputs and outputs. Thus, the issue of regulating AI in biotechnology research remains open. Achieving consensus between the scientific community and policymakers is essential to harness the full potential of AI and its successful implementation in forest biotechnology.

## 5. Perspectives

Trees differ from annual herbaceous plants in several traits, including perennial growth, large size, presence of dormancy phases, and others. Among these, the mechanism of secondary cell wall (wood) formation is not only of interest to researchers, but also has practical importance. Wood is widely used in construction, paper production, biofuels, and other industries, but its composition often differs drastically. Using AI methods to decipher the genetic mechanisms of wood formation with the aim of selecting genotypes with desired properties is an important task. In addition, AI can be used to integrate mono-omics datasets into a unified framework to study complex relationships between them. Such multi-omics analysis will promote better understanding of the biological processes occurring in trees, as well as the development of new breeding strategies [155].

The level and stability of transferred gene expression in transgenic plants are of great importance, especially in perennial woody species. Synthetic promoters that can overcome some limitations of native promoters have recently become more widespread [156]. DL technologies can provide precise promoter identification and enable generation and testing of large numbers of design variants [157]. Another way to increase transgene expression is intron-mediated enhancement [158]. Insertion of intron in the *uidA* gene enhanced its expression in pear leaves and fruits under field conditions [159], and their presence in the *TmDGAT1* gene increased triacylglycerol and total fatty acid accumulation in energycane (*Saccharum* sp.) [160]. Back and Walther [161] used the RF algorithm to identify features responsible for intron-mediated enhancement in Arabidopsis and showed that introns contain information about gene expression level. ML methods can be used to predict intron effectiveness and design intron sequences. Introducing multiple genes into plants is necessary to combine monogenic traits, confer complex polygenic properties, or control metabolic pathways, but such work is challenging in tree species [162]. Applying AI to design multigene vector constructs and ensure optimal, coordinated expression in specific genotypes will simplify the creation of such trees. Moreover, AI can be used to decipher mechanisms underlying unintended effects in transgenic trees: to prevent negative ones or to exploit positive ones, for example, to improve rooting [163] or accelerate flowering [164]. Finally, using ML and DL methods in modeling cultivation of transgenic forest plantations to evaluate complex interactions among genotypes, environmental conditions, and silvicultural practices will allow for the assessment of potential ecological risks at large spatial and temporal scales.

Another promising area of forest biotechnology is the use of synthetic biology. This field combines computer science and engineering principles with life sciences to create plant systems with specified properties [165]. Synthetic biology can be used both for traditional forest traits, including increased productivity and stress tolerance, and to create trees that produce specialized biomass or compounds not found in nature [166]. AI can be applied to design such trees with unique properties.

## 6. Conclusions

This work demonstrates that the use of AI methods in forest biotechnology is an active research area. They offer a wide range of opportunities—from optimizing culture media composition for plant growth in vitro through large omics datasets analysis, to identifying individual trees with desired traits from satellite imagery—to create high-yielding, stress-tolerant varieties with specified quality characteristics. Integration of genotype–environment–phenotype interaction data will advance understanding of various biological mechanisms in woody plants and enable development of targeted breeding strategies, leading to more targeted and efficient production of desired genotypes. Addressing existing challenges requires the collaborative efforts of developers, researchers, and policymakers. Ensuring a human-centered approach requires inclusive model training and the support of explainable AI. Despite all the potential of AI, it should be remembered that it is only a complement to human intelligence and cannot replace its experience, creative thinking, and conceptual approach, such as so-called “breeder’s intuition”. Wider adoption of AI in plant science requires publicly available, high-quality datasets, international data format standards, and staff training. The expanding use of AI and the ethical issues that arise require effective regulation. The lack of such regulation and public concerns may delay AI adoption. Ultimately, this technology will contribute to a more sustainable and productive future for forests across the globe.

## Figures and Tables

**Table 1:** A summary of studies applying artificial intelligence methods in plant biotechnology.

Year	Technology	Crop	Aim of the Study	AI Method	Reference
2010	in vitro culture	kiwifruit	shoot multiplication	MLP	[52]
2011	phenotyping	sugarbeet	disease identification	k-NN	[53]
2011	genomic selection	wheat	yield prediction	MLP	[54]
2012	genomics	wheat	genome analysis	SVM	[55]
2017	interactome	rice	protein–protein interactions	RF	[56]
2018	marker-assisted selection	potato	drought tolerance	RF	[57]
2018	metabolomics	alfalfa	abiotic and biotic stress	k-means	[58]
2019	epigenomics	rice	methylation site prediction	CNN	[59]
2019	genome-wide association study	soybean	yield, protein, oil, moisture, and height prediction	CNN	[60]
2020	proteomics	grapevine	protein identification	RNN	[61]
2021	genetic modification	tobacco, maize	synthetic promoter design	CNN	[62]
2021	genome editing	soybean, maize, sorghum, wheat	identification of sgRNA on-target activity	CNN	[63]

**Table 2:** Examples of applied artificial intelligence methods in forest biotechnology.

Technique	Sub-Division	Plant Species	Application	Method	Reference
Plant tissue culture	cell culture	*Coryllus avellana*	paclitaxel biosynthesis	ANFIS	[64]
	organogenesis	*Pinus taeda*	mineral composition optimization	MLP	[65]
	somatic embryogenesis	*Pinus radiata*	somatic embryo analysis	CNN	[66]
	synthetic seeds	*Crataegus monogyna*	synthetic seed regeneration	DT, GP, MLP, RF, XGBoost	[67]
Genome modification	genetic transformation	*Populus tremula* × *P. tremuloides*	drought tolerance	GMM, k-means	[68]
	genome editing	*Populus trichocarpa*	lignin modification	ML algorithms	[69]
Molecular markers	genetic identification	*Fagus sylvatica*, *Quercus robur*	geographic origin	GPR-D, GPR-F, FFNN	[70]
	genome-wide association study	*Populus tomentosa*	photosynthetic efficiency	XGBoost, RF, SVR, LASSO, KNN	[71]
	genomic selection	*Eucalyptus globulus*	essential oil content, growth, and stem quality	CNN, MLP	[72]
Omics technologies	genomics	*Populus alba* × *P. tremula*	allele-specific gene expression	XGBoost	[73]
	epigenomics	*Populus balsamifera*	trait–methylome association	FFNN	[74]
	transcriptomics	*Populus* sp.	drought-responsive gene identification	SVM, Information Gain, Information Gain Ratio	[75]
	proteomics	*Phyllostachys edulis*	orphan gene identification	RF, SVM, RNN, CNN + RNN, LSTM, CNN + LSTM, GRU, CNN + GRU, Transformer, CNN + Transformer	[76]
	metabolomics	*Populus trichocarpa*	metabolome under drought and endophyte inoculation	LR, KNN, DT	[77]
Phenomics	microphenotyping	~40 species	stomata identification	CNN	[78]
	ground phenotyping	*Betula luminifera*	drought stress identification	five CNN models	[79]
	aerial phenotyping	*Castanea sativa*	disease symptom identification	SVM, GNB, LR	[80]

ANFIS: adaptive neuro-fuzzy inference system; CNN: convolutional neural network; DT: decision tree; FFNN: feedforward neural network; GMM: Gaussian mixture model; GNB: Gaussian Naive Bayes; GP: Gaussian process; GPR: Gaussian process regression; GRU: gated recurrent unit; KNNs: k-nearest neighbors; LASSO: least absolute shrinkage and selection operator; LR: logistic regression; LSTM: long short-term memory; MLP: multilayer perceptron; RF: random forest; RNN: recurrent neural network; SVM: support vector machine; SVR: support vector regression; XGBoost: extreme gradient boosting.

## Data Availability

No new data were created or analyzed in this study. Data sharing is not applicable to this article.

## Abbreviations

The following abbreviations are used in this manuscript:

AI	artificial intelligence
ANFIS	adaptive neuro-fuzzy inference system
ANN	artificial neural network
ASE	allele-specific expression
BIRCH	balanced iterative reducing and clustering using hierarchies
BLUP	best linear unbiased prediction
BPNN	back propagation neural network
BRR	Bayesian ridge regression
CatBoost	categorical gradient boosting
CNN	convolutional neural network
CV	computer vision
DL	deep learning
DNN	deep neural network
DT	decision tree
FFNN	feedforward neural network
FOA	fruit fly optimization algorithm
GA	genetic algorithm
GBDT	gradient boosting decision tree
GBLUP	genomic best linear unbiased prediction
GFP	green fluorescent protein
GMM	Gaussian mixture model
GNB	Gaussian Naive Bayes
GNN	graph neural network
GP	Gaussian process
GPP	gross primary productivity
GPR	Gaussian process regression
GRNN	generalized regression neural network
GRU	gated recurrent unit
GS	genomic selection
GWAS	genome-wide association study
HTP	high-throughput phenotyping
IAA	indole acetic acid
KNN	k-nearest neighbors
LASSO	least absolute shrinkage and selection operator
LDA	linear discriminant analysis
LGB	light gradient boosting
LR	logistic regression
LSTM	long short-term memory
ML	machine learning
MLP	multilayer perceptron
NDVI	normalized difference vegetation index
NIR	near-infrared
QTL	quantitative trait loci
PLS-DA	partial least squares discriminant analysis
RF	random forest
RGB	red–green–blue
RKHS	reproducing kernel Hilbert space regression
RNN	recurrent neural network
SAM	spatial attention module
SM-GK	single-environment, main genotypic effect model with a Gaussian kernel
SNP	single nucleotide polymorphism
SSR	simple sequence repeat
SVM	support vector machine
SVR	support vector regression
TIR	thermal infrared
UAV	unmanned aerial vehicle
UMAP	uniform manifold approximation and projection
XGBoost	extreme gradient boosting
